# Tissue characterization using cardiac magnetic resonance imaging and response to cardiac resynchronization therapy

**DOI:** 10.1093/europace/euaf043

**Published:** 2025-04-10

**Authors:** Se-Eun Kim, Jaewon Oh, Yoo Jin Hong, Daehoon Kim, Hee Tae Yu, Chan Joo Lee, Tae-Hoon Kim, Jae-Sun Uhm, Boyoung Joung, Hui-Nam Pak, Moon-Hyoung Lee, Young Jin Kim, Seok-Min Kang

**Affiliations:** Department of Cardiology, Wonju Severance Christian Hospital, Yonsei University Wonju College of Medicine, Wonju, Republic of Korea; Division of Cardiology, Severance Cardiovascular Hospital, Yonsei University College of Medicine, Seoul, Republic of Korea; Department of Radiology, Yonsei University College of Medicine, Seoul, Republic of Korea; Division of Cardiology, Severance Cardiovascular Hospital, Yonsei University College of Medicine, Seoul, Republic of Korea; Division of Cardiology, Severance Cardiovascular Hospital, Yonsei University College of Medicine, Seoul, Republic of Korea; Division of Cardiology, Severance Cardiovascular Hospital, Yonsei University College of Medicine, Seoul, Republic of Korea; Division of Cardiology, Severance Cardiovascular Hospital, Yonsei University College of Medicine, Seoul, Republic of Korea; Division of Cardiology, Severance Cardiovascular Hospital, Yonsei University College of Medicine, Seoul, Republic of Korea; Division of Cardiology, Severance Cardiovascular Hospital, Yonsei University College of Medicine, Seoul, Republic of Korea; Division of Cardiology, Severance Cardiovascular Hospital, Yonsei University College of Medicine, Seoul, Republic of Korea; Division of Cardiology, Severance Cardiovascular Hospital, Yonsei University College of Medicine, Seoul, Republic of Korea; Department of Radiology, Yonsei University College of Medicine, Seoul, Republic of Korea; Division of Cardiology, Severance Cardiovascular Hospital, Yonsei University College of Medicine, Seoul, Republic of Korea

**Keywords:** Cardiac magnetic resonance imaging, Cardiac resynchronization therapy, Tissue characterization, Mapping value, Late gadolinium enhancement

## Abstract

**Aims:**

Cardiac magnetic resonance (CMR) imaging for tissue characterization offers valuable insights for risk stratification among patients with cardiomyopathy. This study aimed to assess the prognostic value of CMR-based tissue characterization in predicting response to cardiac resynchronization therapy (CRT) in patients with non-ischaemic cardiomyopathy (NICM).

**Methods and results:**

Retrospective analysis was performed on CMR data from NICM patients before CRT implantation. Various CMR parameters, including the late gadolinium enhancement (LGE), native T1, T2, and extracellular volume (ECV), were analysed. Among the 101 patients (mean age: 66 years, male: 52.5%), 72 (71.3%) were CRT responders. The CRT responders had lower LGE burden (13.1 vs. 35.3%, *P* < 0.001), native T1 (1334.5 vs. 1371.6 ms, *P* = 0.012), T2 (42.2 vs. 45.7 ms, *P* < 0.001), and ECV (30.8 vs. 36.8%, *P* < 0.001) compared with CRT non-responders. After adjusting for other risk factors, LGE burden ≤ 20% [odds ratio (OR): 22.61, 95% confidence interval (CI): 4.73–176.68, *P* < 0.001], ECV ≤ 34% (OR: 15.93, 95% CI: 3.01–115.13, *P* = 0.002), and T2 ≤ 45 ms (OR: 8.10, 95% CI: 1.82–43.75, *P* = 0.008) were identified as predictors of good CRT response and favourable clinical outcomes (log-rank *P* < 0.001).

**Conclusion:**

Cardiac magnetic resonance-based tissue parameters effectively predict CRT response and clinical outcomes in patients with NICM, independently of conventional predictors.

## Graphical Abstract

**Graphical Abstract:**
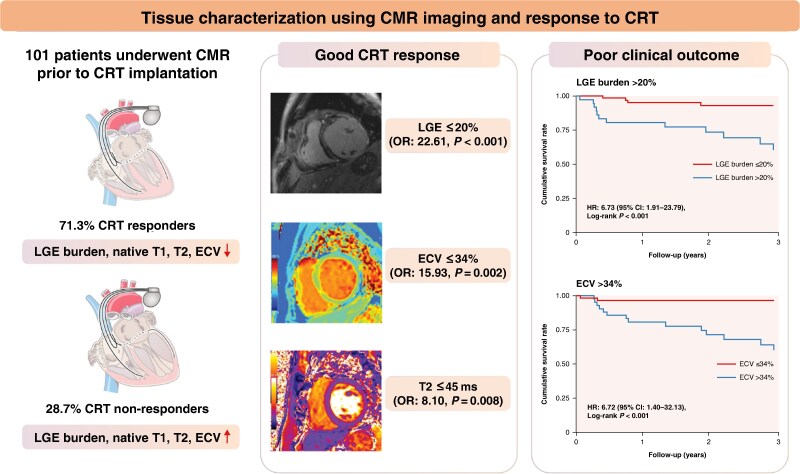
Cardiac magnetic resonance tissue parameters as predictors for CRT response in NICM. Among 101 patients who underwent CMR before CRT implantation, 71.3% were CRT responders, who had lower LGE burden, baseline T1, T2, and ECV values. In contrast, 28.7% were CRT non-responders, who had higher LGE burden, baseline T1, T2, and ECV values. Late gadolinium enhancement burden ≤ 20%, ECV ≤ 34%, and T2 ≤ 45 ms were independent predictors of good CRT response and were associated with favourable clinical outcomes. CMR, cardiac magnetic resonance; CRT, cardiac resynchronization therapy; ECV, extracellular volume; LGE, late gadolinium enhancement; NICM, non-ischaemic cardiomyopathy.

**What’s new?**

- The role of cardiac magnetic resonance (CMR) tissue parameters in predicting cardiac resynchronization therapy (CRT) response is limited to late gadolinium enhancement (LGE), while the role of other CMR parameters has remained uncertain.
- This study sheds light on this issue by demonstrating that CRT non-responders have higher LGE burden, native T1, T2, and extracellular volume values than CRT responders.
- Cardiac magnetic resonance tissue parameters emerged as relevant predictors of poor CRT response and adverse clinical outcomes.
- Using CMR to evaluate the tissue characteristics can help predict CRT response and clinical outcomes in patients with dilated cardiomyopathy.

## Introduction

Mechanical dyssynchrony is a prevalent feature in heart failure (HF) patients, impacting atrioventricular, interventricular, and intraventricular coordination.^1,2^ Cardiac resynchronization therapy (CRT) has emerged as a cornerstone intervention for symptomatic HF patients with reduced ejection fraction and prolonged QRS duration.^1–3^ Large clinical trials have shown that CRT implantation reduces all-cause mortality and HF hospitalization rates while improving the patient symptoms or quality of life.^4,5^ Nonetheless, individual responses to CRT vary. The well-known predictors of CRT response include wide QRS duration, left bundle branch block (LBBB) QRS morphology, female sex, and non-ischaemic cardiomyopathy (NICM).^1–3^ Several imaging modalities have emerged to assess the factors influencing CRT efficacy.^6,7^ Transthoracic echocardiography (TTE) remains a primary diagnostic tool, yet CMR imaging has been highlighted as a promising alternative. Cardiac magnetic resonance offers more accurate cardiac anatomy, function, and myocardial tissue characteristics than TTE. Cardiac magnetic resonance-derived late gadolinium enhancement (LGE) serves as a valuable marker of myocardial fibrosis, while advances in mapping techniques allow parameters such as native T1, T2, and extracellular volume (ECV) to provide additional insights into the myocardium.^8–10^ Numerous studies have demonstrated the association between LGE or mapping values and prognosis, emphasizing the importance of CMR-based tissue characterization in risk stratification for cardiomyopathy.^9–12^ Since the association between the presence of LGE and response to CRT was first reported in 2012,^13^ a large body of literature has shown that the presence of LGE is an independent predictor of response to CRT.^14–18^ However, the role of CMR mapping values (T1, T2, and ECV) in predicting CRT response has been less studied.^19^

This study aimed to examine whether CMR tissue parameters, including LGE, T1, T2, and ECV values, can serve as prognostic factors when assessing CRT response among patients with NICM and whether subsequent clinical events can be reliably predicted.

## Methods

### Study design and populations

This retrospective, observational study was conducted at a single tertiary centre in South Korea. Patients diagnosed with NICM who underwent CRT implantation between January 2014 and September 2022 were included. The diagnosis of NICM was established based on the following criteria: left ventricular dilatation with reduced left ventricular systolic function [left ventricular ejection fraction (LVEF) < 40%] without any definite identifiable cause for left ventricular dysfunction. Cardiac resynchronization therapy implantation was performed in patients with a wide QRS with an LVEF ≤ 35%, as per guidelines.^1,2^ Patients who underwent CMR imaging within 1-year preceding CRT implantation and TTE within at least 3-month post-CRT implantation were included. Patients lacking available mapping images from CMR and those who did not undergo TTE during a 3-month follow-up period post-CRT implantation were excluded (see Supplementary material online, *Figure S1*). All patients underwent either coronary angiography or computed tomography coronary angiography to confirm the absence of coronary artery disease. Demographic information, clinical data, 12-lead electrocardiogram results, and laboratory data were retrieved from the electronic medical records. Cardiac resynchronization therapy implantation was performed per established guidelines.^1–3^

This study was approved by the Institutional Review Board of Yonsei University Health System (approval number: 4-2023-0237) and conducted in compliance with the principles of the Declaration of Helsinki. Given the study’s retrospective design, the requirement for informed consent was waived.

### Transthoracic echocardiography and cardiac magnetic resonance imaging

Transthoracic echocardiography was performed using a standard ultrasound machine (Vivid 7 or E9, GE Medical Systems, Wauwatosa, WI, USA; Philips iE33 or Epiq7, Philips Healthcare, the Netherlands) equipped with a 2.5–3.5 MHz probe. Standard echocardiographic measurements were obtained according to the recommended guidelines of the American Society of Echocardiography.^20^ Left ventricular end-systolic volume (LVESV) and LVEF were measured using the biplane method of the disks from standard apical four-chamber and two-chamber views.

Cardiac magnetic resonance was conducted using a 3 T MR scanner (3T, Prisma Fit, Siemens Healthineers) equipped with a 30-channel array body coil and electrocardiogram gating. The cine, native T1, T2, post-T1 mapping, and LGE sequences were obtained. Detailed acquisition and analysis methods for each image are described in Supplementary material online, *Methods*. The reference values for native T1, ECV, and T2 were obtained using the same CMR protocol and were as follows: 1219.0 ± 29.1 ms, 25.7 ± 2.4%, and 39.6 ± 2.0 ms, respectively. Two investigators (Y.J.H. and Y.J.K., with over 15 years of experience in cardiovascular radiology) analysed all images.

### Cardiac resynchronization therapy response and clinical outcomes

The change in LVESV and LVEF was calculated using TTE before and at least 3 months after CRT implantation. The response to CRT is defined as either a decrease in LVESV of at least 15% on follow-up TTE.^21^

The composite outcome including HF admission or cardiovascular death was determined to evaluate the association of CMR parameters with clinical outcomes.

### Statistical analysis

The patients were initially categorized into two groups based on their responses to CRT. Continuous variables are expressed as the mean ± SD, while categorical variables as frequencies (%). Student’s *t*-test or Fisher’s exact test was used to compare the parameters between the two groups. Paired *t*-tests were used to compare the variables before and after CRT implantation. The factors with a non-normal distribution are expressed as the medians [interquartile range (IQR)] and were compared using the Wilcoxon rank-sum test. Receiver operating characteristic (ROC) curve analysis and the area under the ROC curve (AUC) were used to calculate the predictive and cut-off values of LGE burden, native T1, T2, and ECV for the CRT response. The univariate and multivariate logistic regression analyses were conducted to evaluate the association between CMR parameters and CRT response. Variables in the model used for multivariate analysis are those that meet *P* < 0.01 in univariate analysis. Kaplan–Meier survival analyses were performed to compare the clinical outcomes, while the differences among the groups were compared using log-rank tests. Multivariable Cox proportional analyses were used to identify the prognostic factors for clinical outcomes. A two-sided *P*-value of <0.05 was considered statistically significant. Statistical analyses were conducted using functions including ‘roc’, ‘glm’, and ‘cox’ in R software (version 4.0.2, R Foundation for Statistical Computing, Vienna, Austria).

## Results

### Clinical characteristics of the study population

Of the 357 patients who underwent CRT implantation during the study period, 101 who met the inclusion criteria were enrolled (see Supplementary material online, *Figure S1*). The baseline characteristics of the study population are presented in *Table 1*. The median duration of follow-up echocardiography was 178 (IQR: 160–211) days. Among the patients, 72 (71.3%) were classified as CRT responders, while 29 (28.7%) as CRT non-responders. Notably, only three patients in the CRT response group underwent CRT pacemaker implantation. There were no significant differences between the two groups in terms of age, sex, clinical history, and history of HF medications, except beta-blockers. However, a higher proportion of CRT responders presented with LBBB (97.2 vs. 62.1%, *P* < 0.001) and longer QRS duration (167 ± 17 vs. 156 ± 17 ms, *P* = 0.006). Prior to CRT implantation, no differences were observed in the New York Heart Association classification between the two groups. Additionally, no differences were found in the LVESV or LVEF as measured by TTE. However, the left atrial volume index (LAVI), right ventricular systolic pressure, and E/e′ ratio were found to be higher in the CRT non-responders.

**Table 1:** Baseline characteristics of patients according to CRT response status

	Non-responder	Responder	*P-*value
	(*n* = 29)	(*n* = 72)	
Age, years	66.6 ± 11.3	65.5 ± 11.4	0.662
Male (%)	19 (65.5)	34 (47.2)	0.148
Hypertension (%)	8 (27.6)	23 (31.9)	0.848
Diabetes mellitus (%)	9 (31.0)	25 (34.7)	0.903
Chronic kidney disease (%)	4 (13.8)	9 (12.5)	1.000
AF/AFL (%)	9 (31.0)	12 (16.7)	0.181
LBBB (%)	18 (62.1)	70 (97.2)	<0.001
QRS duration, ms	156 ± 17	167 ± 17	0.006
Pre-NYHA classification			1.000
2 (%)	13 (44.8)	34 (47.2)	
3 (%)	16 (55.2)	38 (52.8)	
Medications			
ACEI/ARBs (%)	8 (27.6)	20 (27.8)	1.000
Sacubitril/valsartan (%)	20 (69.0)	52 (72.2)	0.933
Beta-blockers (%)	24 (82.8)	70 (97.2)	0.031
MRAs (%)	23 (79.3)	63 (87.54)	0.461
Loop diuretics (%)	26 (89.7)	63 (87.5)	1.000
Ivabradine (%)	10 (34.5)	24 (33.3)	1.000
SGLT2 inhibitor (%)	2 (6.9)	7 (9.7)	0.948
Echocardiographic parameters			
LVESV, mL	138.1 (114.18–205.2)	140.8 (111.73–198.1)	0.770
LVEF, %	25.0 (20.0–30.0)	24.0 (20.0–29.0)	0.833
LAVI, mL/m^2^	60.0 (44.0–70.9)	40.0 (31.5–51.8)	0.001
RVSP, mmHg	40.5 (29.8–54.5)	25.0 (22.0–36.0)	0.002
E/e′	26.7 (14.8–31.8)	15.8 (11.6–18.9)	<0.001
Implanted CRT type			0.640
CRT-D (%)	29 (100)	69 (95.8)	
CRT-P (%)	0 (0)	3 (3.9)	
Post QRS duration, ms	150 ± 18	147 ± 21	0.405
Biventricular pacing (%)	96 ± 4	98 ± 2	0.058

Data are expressed as the mean ± standard deviation, median (IQR), and absolute numbers (%).

ACEI, angiotensin-converting enzyme inhibitor; AF, atrial fibrillation; AFL, atrial flutter; ARB, angiotensin receptor blocker; CRT, cardiac resynchronization therapy; CRT-D, cardiac resynchronization therapy defibrillator; CRT-P, cardiac resynchronization therapy pacemaker; NYHA, New York Heart Association; MRAs, mineralocorticoid receptor antagonists; LAVI, left atrial volume index; LBBB, left bundle branch block; LVEDV, end-diastolic volume of the left ventricle; LVEF, left ventricular ejection fraction; LVESV, end-systolic volume of the left ventricle; RVSP, right ventricular systolic pressure; SGLT2, sodium-glucose cotransporter-2.

### Cardiac magnetic resonance parameters of the study population

Regarding baseline CMR parameters, no significant differences were observed between the CRT responders and non-responders in the left ventricle volume and systolic function (*Table 2*). However, CRT responders exhibited significantly lower right ventricular end-diastolic volume (134.8 vs. 169.3 mL, *P* = 0.014) and right ventricular end-systolic volume (77.8 vs. 116.8 mL, *P* = 0.020) compared with CRT non-responders. In terms of the tissue characterization parameters, CRT responders demonstrated significantly lower values for LGE burden (13.1 vs. 35.3%, *P* < 0.001), native T1 (1334.5 vs. 1371.6 ms, *P* = 0.012), T2 (42.2 vs. 45.7 ms, *P* < 0.001), and ECV (30.8 vs. 36.8%, *P* < 0.001) than non-responders.

**Table 2:** CMR parameters according to CRT response status

	Non-responder	Responder	*P-*value
	(*n* = 29)	(*n* = 72)	
LVEDV, mL	258.3 (223.2–321.9)	258.2 (210.4–306.0)	0.755
LVESV, mL	201.7 (167.0–243.5)	194.7 (158.0–247.3)	0.666
LVSV, mL	60.2 ± 20.6	59.0 ± 18.2	0.776
LVEF, %	23.5 ± 9.0	23.2 ± 7.6	0.845
RVEDV, mL	169.3 (136.5–211.3)	134.8 (112.2–174.7)	0.014
RVESV, mL	116.8 (69.5–164.6)	77.8 (57.8–108.4)	0.020
RVSV, mL	57.0 ± 20.2	55.1 ± 16.4	0.633
RVEF, %	35.1 ± 15.1	40.2 ± 13.1	0.092
LGE burden, %	35.3 (23.0–49.0)	13.1 (8.8–18.8)	<0.001
Native T1, ms	1371.6 (1339.0–1419.8)	1334.5 (1304.5–1389.9)	0.012
ECV, %	36.8 (35.3–39.6)	30.8 (28.3–34.3)	<0.001
T2, ms	45.7 (42.9–47.3)	42.2 (40.8–44.0)	<0.001

Data are expressed as the mean ± standard deviation and median (IQR).

CMR, cardiac magnetic resonance; CRT, cardiac resynchronization therapy; ECV, extracellular volume; LGE, late gadolinium enhancement; LVEDV, end-diastolic volume of the left ventricle; LVEF, left ventricular ejection fraction; LVESV, end-systolic volume of the left ventricle; LVSV, left ventricular systolic volume; RVEDV, end-diastolic volume of the right ventricle; RVEF, left ventricular ejection fraction; RVESV, end-systolic volume of the right ventricle; RVSV, right ventricular systolic volume.

### Predictors of cardiac resynchronization therapy response

**Figure 1:**
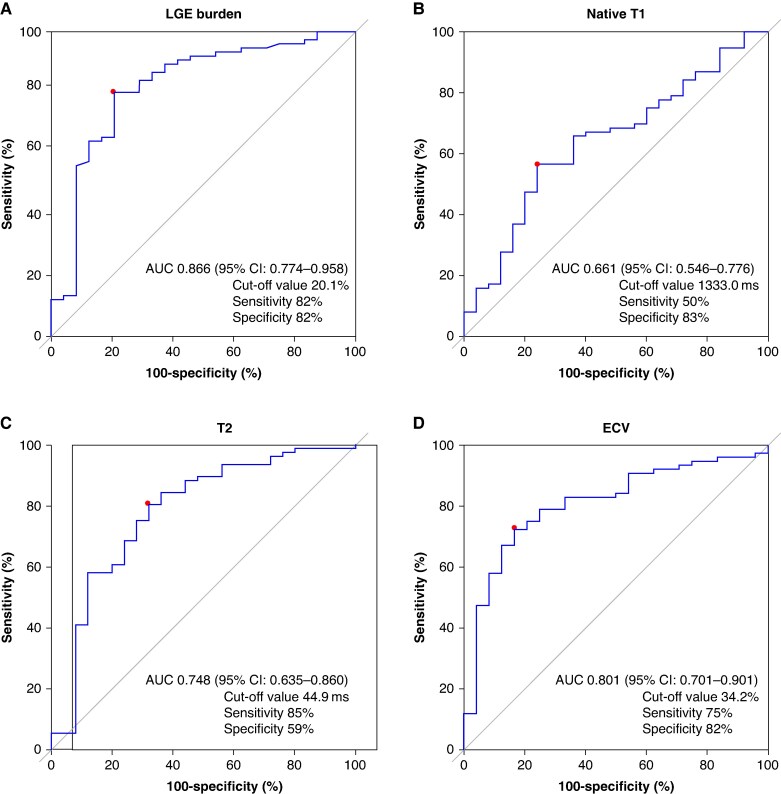
Receiver operating characteristic curves displaying CMR parameters as predictors of CRT response. AUC, area under the ROC curve; CI, confidence interval; CRT, cardiac resynchronization therapy; ECV, extracellular volume; LGE, late gadolinium enhancement; ROC, receiver operating characteristics.

The cut-off values for predicting CRT response were determined using ROC curve analysis (*Figure 1*). The AUC values for LGE burden, native T1, T2, and ECV were 0.866 [95% confidence interval (CI): 0.774–0.958], 0.661 (95% CI: 0.546–0.776), 0.748 (95% CI: 0.635–0.860), and 0.801 (95% CI: 0.701–0.901), respectively. The cut-off value for LGE burden was 20.1% with a sensitivity of 82% and specificity of 82%. The cut-off value, sensitivity, and specificity were 1333.0 ms, 50%, and 83%, respectively, for native T1; 44.9 ms, 85%, and 59%, respectively, for T2; and 34.2%, 75%, and 82%, respectively, for ECV. *Table 3* and Supplementary material online, *Table S1* present the predictors of CRT response including the clinical characteristics and tissue characterization parameters identified by CMR. Following patient stratification based on the cut-off values of tissue characterization parameters, an LGE burden ≤ 20% [odds ratio (OR): 22.61, 95% CI: 4.73–176.68, *P* < 0.001], ECV ≤ 34% (OR: 15.93, 95% CI: 3.01–115.13, *P* = 0.002), and T2 ≤ 45 ms (OR: 8.10, 95% CI: 1.82–43.75, *P* = 0.008) were identified as relevant predictors of reduced likelihood of CRT response. The presence of LGE was also a significant predictor for CRT response (OR: 5.73, 95% CI: 1.13–42.55, *P* = 0.034). Of these, ECV ≤ 34 ms and LGE burden ≤ 20% were more useful predictors of CRT response than the presence of LGE or T2 ≤ 45 ms (see Supplementary material online, *Table S2*).

**Table 3:** Predictors of CRT response

	Model 1			Model 2			Model 3			Model 4			Model 5		
	OR	95% CI	*P*-value	OR	95% CI	*P*-value	OR	95% CI	*P*-value	OR	95% CI	*P*-value	OR	95% CI	*P*-value
LBBB	9.00	0.71–215.20	0.119	17.03	0.80–980.19	0.106	8.02	0.69–187.83	0.131	18.98	1.16–800.55	0.070	7.72	0.56–243.39	0.169
QRS duration ≥ 150 ms	3.31	0.68–16.50	0.133	2.08	0.28–16.00	0.471	2.36	4.57–11.33	0.284	1.87	0.35–9.64	0.453	1.86	0.33–9.95	0.498
Presence of LGE	5.73	1.13–42.55	0.034												
LGE burden ≤ 20%				22.61	4.73–176.68	<0.001									
Native T1 ≤ 1333 ms							2.56	0.61–12.47	0.212						
ECV ≤ 34%										15.93	3.01–115.13	0.002			
T2 ≤ 45 ms													8.10	1.82–43.75	0.008

All models are adjusted for right ventricular end-systolic volume measured by cardiac magnetic resonance imaging, baseline right ventricular systolic blood pressure, E/e′, left atrial volume index, beta-blocker prescription, LBBB, QRS duration (≥150 ms), biventricular pacing rate, presence of LGE (Model 1), cut-off value for LGE burden (Model 2), cut-off value for baseline T1 (Model 3), cut-off value for ECV (Model 4), and cut-off value for T2 (Model 5).

CI, confidence interval; CRT, cardiac resynchronization therapy; ECV, extracellular volume; LBBB, left bundle branch block; LGE, late gadolinium enhancement; OR, odds ratio.

### Predictors of cardiac resynchronization therapy response in patients with left bundle branch block and a QRS duration ≥ 150 ms

Among the patients, 75 showed favourable electrocardiographic factors, such as LBBB and a QRS duration ≥ 150 ms, both considered a Class I indication for CRT implantation according to the current HF guidelines. Of these patients, 13 (17.3%) were classified as CRT non-responders. No differences were found in clinical characteristics or medical history between CRT responders and non-responders. However, the echocardiographic parameters revealed higher LAVI and E/e′ values in CRT non-responders (see Supplementary material online, *Table S3*). Among patients with Class I indication for CRT, no significant differences were observed in CMR-based chamber size or function between the CRT responders and non-responders. Moreover, no significant differences were observed in the native T1 and T2 values between the two groups; however, the CRT responders had significantly lower LGE burden (13.3 vs. 38.9%, *P* < 0.001) and ECV (30.3 vs. 37.4%, *P* < 0.001) than the CRT non-responders (see Supplementary material online, *Table S4*). In patients with a Class I indication for CRT, the cut-off value for LGE burden was 24% (AUC: 0.805, 95% CI: 0.618–0.992), while the cut-off value for ECV was 34.2% (AUC: 0.8133, 95% CI: 0.669–0.957) (see Supplementary material online, *Figure S2*). Following adjustment for E/e′, an LGE burden ≤ 24% (OR: 11.62, 95% CI: 2.41–69.32, *P* = 0.003) and ECV ≤ 34% (OR: 11.22, 95% CI: 1.94–91.68, *P* = 0.010) emerged as significant predictors of good CRT response in this patient group (see Supplementary material online, *Table S5*). Meanwhile, 26 patients did not meet the Class I indication for CRT, either they were LBBB but QRS duration < 150 ms or not LBBB but QRS duration ≥ 150 ms. There was no difference in QRS duration in these patients and no difference in echocardiographic parameters including baseline LVEF (see Supplementary material online, *Table S6*). However, the CRT responders had significantly lower LGE burden (9.7 vs. 34.3%, *P* < 0.001) and T2 (43.9 vs. 47.2 ms, *P* = 0.009) than the CRT non-responders (see Supplementary material online, *Table S7*). Late gadolinium enhancement burden and ECV were also associated with CRT response in these patients, too (see Supplementary material online, *Table S8*).

### Clinical outcomes

During a median follow-up of 2.5 years (IQR: 1.3–4.1 years), 19 composite events occurred. Among these, 14 patients were readmitted due to worsening of HF and 7 died due to cardiovascular causes. Supplementary material online, *Table S9* describes the baseline characteristics and CMR parameters of the study population according to the occurrence of composite events. Patients who experienced events had significantly higher mapping parameters than those who did not. Among the CRT non-responders, 15 patients (51.7%) experienced HF-related admission or cardiovascular death. In contrast, among the CRT responders, four patients (5.6%) experienced these composite events. Remarkably, CRT responders had a significantly lower occurrence of events than the CRT non-responders (log-rank *P* < 0.001) (see Supplementary material online, *Figure S3*). These findings suggest that CRT responders experienced a more favourable clinical outcome, with a lower incidence of composite events than the non-responders.

**Figure 2:**
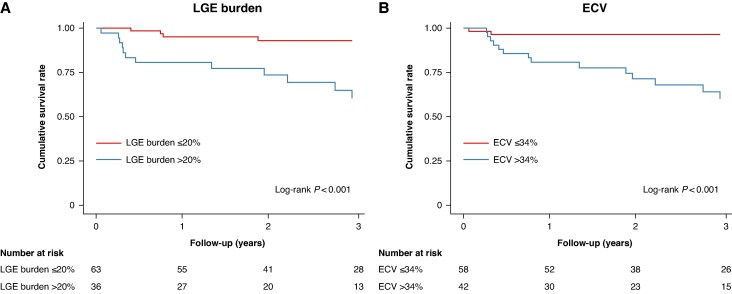
Clinical outcomes according to ECV and LGE burden. ECV, extracellular volume; LGE, late gadolinium enhancement.

When patients were stratified based on the cut-off value of LGE burden (20%) or ECV (34%), those with an LGE burden > 20% or ECV > 34% experienced significantly worse clinical outcomes than those with an LGE burden ≤ 20% or ECV ≤ 34% (log-rank *P* < 0.001; *Figure 2*). After adjustment, an LGE burden > 20% [hazard ratio (HR): 6.73, 95% CI: 1.91–28.79, *P* = 0.003] and ECV > 34% (HR: 6.72, 95% CI: 1.40–32.13, *P* = 0.017) were identified as independent prognostic factors for poor clinical outcomes in patients with NICM who underwent CRT implantation (see Supplementary material online, *Table S10*). The categorization of patients based on the LGE burden and ECV cut-off values demonstrated a gradual increase in the incidence of clinical events and a decrease in the rate of CRT response (*P* for trend < 0.001; Supplementary material online, *Table S11*). The Kaplan–Meier curve showed that significantly fewer events occurred in patients with an LGE burden ≤ 20% or ECV ≤ 34 ms than in those with an LGE or ECV above the cut-off value (log-rank *P* < 0.001; Supplementary material online, *Figure S4*).

## Discussion

This study demonstrates the value of CMR-based tissue characterization in effectively predicting CRT response in individuals with NICM. Cardiac resynchronization therapy non-responders had higher LGE burden, native T1, T2, and ECV values than CRT responder. Additionally, an LGE burden or ECV independently predicted poor CRT response and adverse clinical outcomes. In patients with a Class I indication for CRT implantation according to the current HF guidelines (LBBB and a QRS duration ≥ 150 ms), CRT non-responders had higher LGE burden and ECV values than the CRT responders, with both parameters serving as predictors of CRT response.

Cardiac magnetic resonance plays a pivotal role in identifying and characterizing myocardial fibrosis.^8,9^ While previous studies predominantly focused on LGE imaging, recent advancements in mapping techniques have emerged as superior in reflecting diffuse myocardial fibrosis than LGEs, enhancing their clinical utility.^8,10,22^ Specifically, T1 mapping detects subtle and diffuse changes in the myocardial tissue composition. However, the native T1 technique can be affected by technical factors such as the magnetic field or contrast agent. In contrast, the ECV, derived from the T1 relaxation time, compensates for these technical influences, providing a more accurate reflection of alterations in myocardial tissue composition than native T1 values. As a result, ECV has been reported as an earlier marker and potentially superior for prognostication in NICM patients.^23^ T2 mapping, another CMR technique, offers insights into myocardial tissue characteristics such as oedema and inflammation.^24^ Increased T2 values in patients with myocardial disease often precede the manifestation of symptoms, changes in ejection fraction, and irreversible myocardial remodelling, underscoring their potential as early detection markers of myocardial damage.^25^

Myocardial fibrosis is one of the crucial pathophysiological mechanisms of HF, representing a significant aspect of cardiac remodelling.^26^ This factor also holds relevance for CRT response, because even patients with favourable factors can exhibit a poor CRT response if the myocardium is nonviable, scarred, or inflamed.^25^ Several studies have investigated the correlation between CRT response and CMR imaging findings.^27^ Notably, myocardial LGE in specific regions or substantial LGE burden has been linked to suboptimal CRT response and clinical outcomes.^14,16,18,28,29^ Native T1 and ECV have also demonstrated potential as predictive factors for the response to CRT.^30^ However, these studies included a small number of NICM patients, fewer than 50, yielding conflicting results regarding the predictive role of native T1 or ECV.^15^ Moreover, a substantial portion of CRT response studies has predominantly focused on evaluating the LGE and mainly included patients with ischaemic cardiomyopathy, thereby limiting their clinical relevance for NICM patients.^31^ Furthermore, a recent study has shown that cardiac inflammation preceding CRT implantation subsequently correlates with CRT response, suggesting that cardiac inflammation may also influence CRT response.^25^ In this context, T2 mapping, a marker of myocardial inflammation, has not yet been studied but is potentially a non-invasive predictor of CRT response. Hence, the clinical value of tissue characterization by CMR to determine the CRT response in patients with NICM warrants further elucidation.

In contrast to previous studies, our study explored the potential of CMR tissue parameters as predictors of CRT response in patients with NICM, with a particular focus on mapping values. Regardless of the presence of LGE, higher ECV and T2 values at baseline were associated with a higher likelihood of unfavourable CRT response. In addition, this study unequivocally demonstrated a significant correlation between mapping values and clinical outcomes. Notably, the patients with both high ECV and LGE burden had the worst prognosis. In this regard, our study serves as a noteworthy contribution, highlighting that tissue characterization utilizing CMR parameters can offer valuable and additional prognostic insights into predicting CRT response and subsequent clinical outcomes in NICM patients.

Cardiac resynchronization therapy non-response is associated with poor survival. Therefore, it is important to identify the characteristics of a favourable CRT response when considering CRT implantation.^1,2,32^ Despite favourable electrical characteristics such as LBBB, some patients fail to exhibit a favourable CRT response, underlining the need to identify additional predictors beyond well-established factors. A recent meta-analysis of various CRT trials confirmed that, in addition to LBBB, CRT implantation in intraventricular conduction delay (IVCD) was associated with a lower risk of HF hospitalization or death, suggesting that IVCD should also be considered a significant factor in recommending CRT.^33^ However, these findings primarily classify CRT candidates based on the electrical characteristics without elucidating why some patients respond well to CRT despite the absence of LBBB or IVCD or why others respond poorly to CRT despite having LBBB or a QRS duration > 150 ms. Other studies utilizing echocardiography have proposed its utility in selecting patients with unfavourable electrical characteristics. By identifying electromechanical dyssynchrony using echocardiographic parameters such as two-dimensional images, myocardial strain images, and myocardial work, these studies have demonstrated the ability to identify patients poised to benefit from CRT.^6,7,34^ However, ∼10% of patients with favourable echocardiographic parameters still responded poorly to CRT. This finding highlights the unmet need to explore additional mechanisms influencing CRT response beyond relying solely on the electrical characteristics from an electrocardiogram or electromechanical dyssynchrony assessed using TTE.

The present study demonstrated that ECV and T2 values were associated with CRT response, irrespective of electrical characteristics such as LBBB morphology or QRS duration. Interestingly, in our study, 13% of patients with LBBB and a QRS duration ≥ 150 ms (Class I indication) were CRT non-responders. This group showed higher ECV values than CRT responders. Conversely, 10.9% had CRT responses despite not having Class I indications for CRT. They had lower T2 values compared with CRT non-responders. In this context, this novel approach of utilizing myocardial characterization via CMR to predict CRT response introduces a fresh perspective not yet explored in research. By confirming the significant role of myocardial fibrosis alongside electrical or electromechanical dyssynchrony in CRT response, our findings pave the way for future CRT candidate selection strategies incorporating CMR findings rather than solely relying on electrical or electromechanical dyssynchrony criteria.

### Study limitations

This study had some limitations. First, it was conducted on a small population at a single centre, which may limit the generalizability of our findings. However, considering the scarcity of previous studies investigating the relationship between tissue characteristics and CRT response using novel CMR mapping techniques and the predominant focus on patients with ischaemic cardiomyopathy in existing literature, our study, which included solely patients with NICM, provides a representative contribution. Second, the values obtained through TTE may be underestimated if the myocardial border is not clearly visible due to limitations in the acoustic window, which can lead to discrepancies between LVESV values measured on TTE and CMR. In this study, the LVESV measured on TTE was smaller than that measured on CMR, and CRT response was defined based on the change in LVESV measured on TTE, as the CMR was no longer available post-CRT implantation. However, since the CRT response was evaluated using the same technique (TTE), the evaluation of the CRT response remains consistent. Third, the cut-off values presented in this paper are not absolute as they were only identified in this study population and are limited in that no external or internal validation was performed. In addition, the 95% CIs are very wide, indicating high inter-subject variability, resulting in low precision for the association of each parameter with CRT response. Thus, it is necessary to generalize the cut-off value through future studies. Fourth, we did not analyse the relationship between LV lead position and tissue characterization on CMR, which continues to warrant further study. Lastly, this study did not analyse the left ventricular strain or myocardial work, which are emerging predictors of CRT response. Therefore, further research integrating various imaging techniques, including contemporary TTE parameters and CMR with novel mapping techniques, is warranted to comprehensively explore predictive variables for CRT response.

In conclusion, utilizing mapping values (e.g. high ECV or high T2) from CMR to evaluate tissue characteristics has demonstrated significant value in predicting both CRT response and clinical outcomes in patients with NICM. Further research is necessary to corroborate our findings.

## Supplementary Material

euaf043_Supplementary_Data

## Data Availability

All data are available from the corresponding author on request.
