# Supplementary material for: Tissue characterization using cardiac magnetic resonance imaging and response to cardiac resynchronization therapy
Source: Europace. 2025 Apr 10;27(4):euaf043. doi: 10.1093/europace/euaf043 (PMC11982015; doi:10.1093/europace/euaf043)
Supplement: euaf043_Supplementary_Data [file euaf043_supplementary_data.docx]

**Supplemental Material**

**Supplemental Methods**

Acquisition and analysis methods for cardiovascular magnetic resonance imaging 3

**Supplemental Tables**

**Supplemental Table 1** Predictors of CRT response identified in the univariate analysis 5

**Supplemental Table 2** Two models with combination of LGE, ECV, and T2 values for CRT response 7

**Supplemental Table 3** Baseline characteristics according to the CRT response status in patients with LBBB and a QRS duration ≥ 150 ms 8

**Supplemental Table 4** CMR parameters according to the CRT response status in patients with class I indication for CRT 10

**Supplemental Table 5** Predictors of CRT response in patients with class I indication for CRT 12

**Supplemental Table 6** Baseline characteristics according to the CRT response status in patients who do not meet class I indication for CRT 13

**Supplemental Table 7** CMR parameters according to the CRT response status in patients who do not meet class I indication for CRT 15

**Supplemental Table 8** Predictors of CRT response in patients with LBBB and QRS duration < 150 ms or without LBBB and QRS duration ≥ 150 ms 17

**Supplemental Table 9** Baseline characteristics according to HF admission, or cardiovascular death 18

**Supplemental Table 10** Multivariate Cox regression analysis of the HF admission, or cardiovascular death 21

**Supplemental Table 11** Incidence of HF admission, or cardiovascular death in four groups based on the cut-off values of LGE burden and ECV 22

**Supplemental Figures**

**Supplemental Figure 1** Flowchart of the participant selection process 23

**Supplemental Figure 2** ROC curves displaying the LGE burden and ECV as predictors of CRT response in patients with LBBB and a QRS duration ≥ 150 ms 24

**Supplemental Figure 3** Kaplan–Meier curves for HF admission, or cardiovascular death rates according to CRT response 25

**Supplemental Figure 4** Kaplan–Meier curves of HF admission, or cardiovascular death rates in the four groups classified by the cut-off values of LGE burden and ECV 26

**Supplemental Methods.** The acquisition and analysis methods for cardiovascular magnetic resonance imaging

Four- and two-chamber cine images, including one plane, and short axis cine images, including the entire ventricle, were obtained using a retrospective electrocardiogram gating technique with a balanced steady-state free precession (true fast imaging with steady-state precession [TrueFISP]) sequence. Native T1 mapping images were acquired using a modified look-locker inversion-recovery 5(3)3 (MOLLI) sequence in three short-axis planes (apical, mid, and base of the left ventricle) by applying a nonselective inversion pulse (TrueFISP single-shot readout sequence in the mid-diastolic phase). T2 mapping images were acquired using a T2-prepared single-shot TrueFISP sequence along the same three short-axis planes with T1 mapping images. Late gadolinium enhancement (LGE) images were obtained 10 min after the injection of contrast agent (0.2 mmol/kg of gadolinium contrast, gadoterate meglumine [Dotarem], Guerbet). A normal myocardium was represented using a phase-sensitive inversion recovery-prepared TrueFISP sequence with the inversion time adjusted to null. The LGE images covered the entire left ventricle along the same three short-axis planes. A fast low-angle shot sequence with different inversion times (150–650 ms to null) was used to determine the inversion time before LGE imaging. Post-contrast T1 mapping images were acquired 15 min after the injection of contrast agent along the same three short-axis of left ventricular images used for T1 with the scheme ‘4(1)3(1)2’ using three inversion pulses. The hematocrit levels were evaluated immediately before performing cardiac magnetic resonance (CMR).

The Cvi42 MR analysis software (Circle Cardiovascular Imaging Inc.) was used to analyze the CMR data. The ventricular function was evaluated using end-systolic and end-diastolic volumes from short-axis cine images by manually delineating the endocardial and epicardial borders of the left ventricle. The ventricular volume at the end of the systolic and diastolic phases, stroke volume, left ventricular mass, and LVEF were automatically calculated. The global native T1, T2, post-contrast T1, and ECV values were measured in 16 myocardial segments, except for the apical segment. The myocardial edges were excluded using a 10% offset to decrease the partial volume artifact. ECV was measured using the following equation:

ECV (%) = (ΔR1 of myocardium/ΔR1 of the left ventricular blood pool) × (1 − hematocrit) × 100.

The left ventricle blood pool T1 values were measured using a circular region of interest > 10 mm^2^, avoiding the papillary muscle. A motion-corrected T1 map provided by Siemens software was used for this analysis, and a 10% offset method was used to avoid partial volume artifacts. Segments with visible artifacts were excluded from the analysis. LGE was quantified using the 5-standard deviation (SD) method.

**Supplemental Table 1. Predictors of CRT response identified in the univariate analysis**

|  | **Odds Ratio** | **95%**  **confidence interval** | ***P*-value** |
| --- | --- | --- | --- |
| **Female** | 2.12 | 0.88–5.35 | 0.099 |
| **Hypertension** | 1.23 | 0.49–3.34 | 0.668 |
| **Diabetes mellitus** | 1.18 | 0.48–3.08 | 0.723 |
| **Chronic kidney disease** | 0.89 | 0.26–3.54 | 0.861 |
| **AF/AFL** | 0.44 | 0.16–1.23 | 0.113 |
| **RVSP** | 0.96 | 0.93–0.99 | 0.004 |
| **E/e’** | 0.90 | 0.84–0.95 | <0.001 |
| **LAVI** | 0.97 | 0.95–0.99 | 0.002 |
| **Beta-blocker** | 7.29 | 1.47–53.30 | 0.022 |
| **LBBB** | 21.39 | 5.18–146.55 | <0.001 |
| **QRS duration (≥ 150ms)** | 5.69 | 2.10–16.16 | <0.001 |
| **Post QRS duration** | 0.99 | 0.97-1.01 | 0.402 |
| **Biventricular pacing** | 1.21 | 1.03-1.45 | 0.029 |
| **RVESV > 111.7 mL** | 5.38 | 2.15–14.05 | <0.001 |
| **RVEDV > 148 mL** | 3.49 | 1.43–9.10 | 0.008 |
| **Presence of LGE** | 3.14 | 1.14–10.22 | 0.037 |
| **LGE burden ≤ 20 %** | 20.52 | 7.04–70.90 | <0.001 |
| **Native T1 ≤ 1,344 ms** | 4.8 | 1.76–15.51 | 0.004 |
| **ECV ≤ 34 %** | 12.83 | 4.57–42.70 | <0.001 |
| **T2 ≤ 45 ms** | 7.86 | 3.02–21.67 | <0.001 |

AF/AFL, atrial fibrillation/atrial flutter; CRT, cardiac resynchronisation therapy; ECV, extracellular volume; LAVI, left atrium volume index; LBBB, left bundle branch block; LGE, late gadolinium enhancement; RVEDV, end-diastolic volume of right ventricle; RVESV, end-systolic volume of right ventricle; RVSP, right ventricle systolic pressure

**Supplemental Table 2. Two models with combination of LGE, ECV, and T2 values for CRT response**

|  | **Model 1** | | | **Model 2** | | |
| --- | --- | --- | --- | --- | --- | --- |
|  | **OR** | **95% CI** | ***P*-value** | **OR** | **95% CI** | ***P*-value** |
| **Presence of LGE** | 3.19 | 0.50–27.51 | 0.242 |  |  |  |
| **LGE burden ≤ 20%** |  |  |  | 27.23 | 4.12–514.29 | 0.004 |
| **ECV ≤ 34 %** | 7.59 | 1.10–69.36 | 0.049 | 7.06 | 0.83–86.45 | 0.086 |
| **T2 ≤ 45 ms** | 2.11 | 0.36–13.99 | 0.415 | 4.19 | 0.52–51.89 | 0.203 |
| **AUC** | 0.901 | | | 0.945 | | |

* All models are adjusted for right ventricular end-systolic volume measured by cardiac magnetic resonance imaging, baseline right ventricular systolic blood pressure, E/e’, left atrial volume index, beta-blocker prescription, left bundle branch block, QRS duration (≥150 ms), biventricular pacing rate.

AUC, area under curve; CI, confidence interval; CRT, cardiac resynchronisation therapy; ECV, extracellular volume; LGE, late gadolinium enhancement; OR, odds ratio

**Supplemental Table 3. Baseline characteristics according to the CRT response status in patients with LBBB and a QRS duration ≥ 150ms.**

|  | **Non-responder** | **Responder** | ***P*-value** |
| --- | --- | --- | --- |
|  | **(N=13)** | **(N=62)** |  |
| Age (years) | 68.5 ± 11.2 | 65.2 ± 11.8 | 0.348 |
| Male | 10 (76.9%) | 30 (48.4%) | 0.117 |
| Hypertension | 3 (23.1%) | 20 (32.3%) | 0.747 |
| Diabetes mellitus | 6 (46.2%) | 20 (32.3%) | 0.524 |
| Chronic kidney disease | 2 (15.4%) | 7 (11.3%) | 1.000 |
| AF/AFL | 3 (23.1%) | 9 (14.5%) | 0.727 |
| QRS duration, ms | 169 ± 14 | 171 ± 14 | 0.646 |
| Pre-NYHA classification |  |  | 1.000 |
| 2 | 7 (53.8%) | 32 (51.6%) |  |
| 3 | 6 (46.2%) | 30 (48.4%) |  |
| **Medications** | | | |
| ACEI/ARBs | 2 (15.4%) | 17 (27.4%) | 0.578 |
| Sacubitril/valsartan | 11 (84.6%) | 45 (72.6%) | 0.578 |
| Beta-blockers | 12 (92.3%) | 60 (96.8%) | 1.000 |
| MRAs | 11 (84.6%) | 56 (90.3%) | 0.911 |
| Loop diuretics | 12 (92.3%) | 54 (87.1%) | 0.955 |
| Ivabradine | 3 (23.1%) | 21 (33.9%) | 0.666 |
| SGLT2 inhibitor | 2 (15.4%) | 5 (8.1%) | 0.764 |
| **Echocardiographic parameters** | | | |
| LVESV, mL | 149.1 (115.6–211.8) | 142.3 (112.9–206.0) | 0.770 |
| LVEF, % | 28.0 (22.0–30.0) | 24.0 (20.0–28.0) | 0.833 |
| LAVI, mL/m^2^ | 47.0 (44.0–66.1) | 38.4 (31.3–48.5) | 0.001 |
| RVSP, mmHg | 32 (25.0–46.0) | 25.0 (21.0–36.0) | 0.002 |
| E/e’ | 26.7 (13.8–34.6) | 16.0 (11.5–19.0) | <0.001 |
| Post QRS duration, ms | 153 ± 19 | 149 ± 20 | 0.536 |
| Biventricular pacing,% | 97 ± 3 | 98 ± 2 | 0.379 |

Data are expressed as the mean ± standard deviation, median (interquartile range), and absolute numbers (%)

ACEI, angiotensin converting enzyme inhibitor; AF, atrial fibrillation; AFL, atrial flutter; ARB, angiotensin receptor blocker; CRT, cardiac resynchronisation therapy; NYHA, New York Heart Association; MRAs, mineralocorticoid receptor antagonists; LAVI, left atrial volume index; LBBB, left bundle branch block; LVEF, left ventricular ejection fraction; LVESV, end systolic volume of left ventricle; LVSV, left ventricular systolic volume; LVEF, left ventricular ejection fraction; RVSP, right ventricular systolic pressure; SGLT2, sodium-glucose cotransporter-2

**Supplemental Table 4.** **CMR parameters according to CRT response status in patients with class I indication for CRT**

|  | **Non-responder** | **Responder** | | ***P*-value** |
| --- | --- | --- | --- | --- |
|  | **(N=13)** | | **(N=62)** |  |
| LVEDV, mL | 253.7 (237.6–344.8) | | 264.4 (224.7–314.1) | 0.700 |
| LVESV, mL | 201.7 (167.0–248.1) | | 201.9 (164.5–258.1) | 0.850 |
| LVSV, mL | 63.7 ± 21.8 | | 60.5 ± 17.2 | 0.564 |
| LVEF, % | 24.3 ± 10.1 | | 23.1 ± 7.5 | 0.619 |
| RVEDV, mL | 158.9 (120.4–207.5) | | 137.6 (114.4–174.9) | 0.389 |
| RVESV, mL | 78.1 (50.3–141.9) | | 77.8 (57.6–108.0) | 0.839 |
| RVSV, mL | 60.6 ± 21.5 | | 56.9 ± 15.0 | 0.448 |
| RVEF, % | 41.5 ± 17.8 | | 41.3 ± 12.9 | 0.954 |
| LGE burden, % | 38.9 (22.8–44.3) | | 13.3 (9.4–18.9) | <0.001 |
| Native T1, ms | 1339.0 (1309.6–1391.2) | | 1334.5 (1302.5–1384.1) | 0.753 |
| ECV, % | 37.4 (35.1–38.9) | | 30.3 (28.4–33.9) | <0.001 |
| T2, ms | 43.6 (41.6–45.9) | | 41.9 (40.4–43.9) | 0.144 |

Data are expressed as the mean ± standard deviation and median (interquartile range).

CMR, cardiovascular magnetic resonance imaging; CRT, cardiac resynchronisation therapy; ECV, extracellular volume; LBBB, left bundle branch block; LGE, late gadolinium enhancement; LVEDV, end-diastolic volume of the left ventricle; LVESV, end-systolic volume of the left ventricle; LVSV, left ventricular systolic volume; LVEF, left ventricular ejection fraction; RVEDV, end-diastolic volume of the right ventricle; RVESV, end-systolic volume of the right ventricle; RVSV, right ventricular systolic volume; RVEF, left ventricular ejection fraction.

**Supplemental Table 5. Predictors of CRT response in patients with class I indication for CRT**

| **Total**  **(N = 75)** | **Odds ratio^*^**  **[95% confidence interval]** | ***P*-value** |
| --- | --- | --- |
| **Presence of LGE** | 2.20 [0.42-16.69] | 0.378 |
| **LGE burden ≤ 24 %**† | 11.62 [2.41–69.32] | 0.003 |
| **Native T1 ≤ 1,334 ms**† | 0.86 [0.17-4.16] | 0.846 |
| **ECV ≤ 34 %**† | 11.22 [1.94–91.68] | 0.010 |
| **T2 ≤ 45 ms**† | 2.46 [0.46–11.92] | 0.269 |

^*^Adjusted by E/e’

†The cut-off values of LGE burden, native T1, and ECV for predicting CRT response identified by an receiver operating characteristic curve analysis in patients with left bundle branch block and a QRS duration ≥ 150 ms.

CRT, cardiac resynchronisation therapy; ECV, extracellular volume; LGE, late gadolinium enhancement

**Supplemental Table 6. Baseline characteristics according to the CRT response status in patients who do not meet class I indication for CRT.**

|  | | **Non-responder** | | **Responder** | | ***P*-value** |
| --- | --- | --- | --- | --- | --- | --- |
|  | | **(N=16)** | | **(N=10)** | |  |
| Age (years) | | 64.9 ± 11.5 | | 67.2 ± 9.1 | | 0.602 |
| Male | | 9 (56.2%) | | 4 (40.0%) | | 0.687 |
| Hypertension | | 5 (31.2%) | | 3 (30.0%) | | 1.000 |
| Diabetes mellitus | | 3 (18.8%) | | 5 (50.0%) | | 0.214 |
| Chronic kidney disease | | 2 (12.5%) | | 2 (20.0%) | | 1.000 |
| AF/AFL | | 6 (37.5%) | | 3 (30.0%) | | 1.000 |
| QRS duration, ms | | 147 ± 11 | | 143 ± 12 | | 0.428 |
| Pre-NYHA classification | |  | |  | | 0.614 |
| 2 | | 6 (37.5%) | | 2 (20.0%) | |  |
| 3 | | 10 (62.5%) | | 8 (80.0%) | |  |
| **Medications** | | | | | | |
| ACEI/ARBs | | 6 (37.5%) | | 3 (30.0%) | | 1.000 |
| Sacubitril/valsartan | | 8 (53.3%) | | 8 (72.7%) | | 0.774 |
| Beta-blockers | | 12 (75.0%) | | 10 (100.0%) | | 0.246 |
| MRAs | | 12 (75.0%) | | 7 (70.0%) | | 1.000 |
| Loop diuretics | | 14 (87.5%) | | 9 (90.0%) | | 1.000 |
| Ivabradine | | 7 (43.8%) | | 3 (30.0%) | | 0.774 |
| SGLT2 inhibitor | | 0 (0.0%) | | 2 (20.0%) | | 0.269 |
| **Echocardiographic parameters** | | | | | | |
| LVESV, mL | | 132.1 (107.2–199.3) | | 122.4 (86.0–144.0) | | 0.262 |
| LVEF, % | | 24.1 ± 5.6 | | 28.0 ± 6.6 | | 0.123 |
| LAVI, mL/m^2^ | | 71.0 ± 34.8 | | 54.0 ± 26.9 | | 0.200 |
| RVSP, mmHg | | 49.4 ± 17.6 | | 35.3 ± 15.3 | | 0.059 |
| E/e’ | | 25.3 ± 9.5 | | 18.8 ± 11.0 | | 0.145 |
| Post QRS duration, ms | 149 ± 15 | | 158 ± 20 | | 0.449 | |
| Biventricular pacing,% | 96 ± 4 | | 99 ± 1 | | 0.401 | |

Data are expressed as the mean ± standard deviation, median (interquartile range), and absolute numbers (%)

ACEI, angiotensin converting enzyme inhibitor; AF, atrial fibrillation; AFL, atrial flutter; ARB, angiotensin receptor blocker; CRT, cardiac resynchronisation therapy; NYHA, New York Heart Association; MRAs, mineralocorticoid receptor antagonists; LAVI, left atrial volume index; LBBB, left bundle branch block; LVEF, left ventricular ejection fraction; LVESV, end systolic volume of left ventricle; LVSV, left ventricular systolic volume; LVEF, left ventricular ejection fraction; RVSP, right ventricular systolic pressure; SGLT2, sodium-glucose cotransporter-2

**Supplemental Table 7. CMR parameters according to the CRT response status in patients who do not meet class I indication for CRT.**

|  | **Non-responder** | **Responder** | | ***P*-value** |
| --- | --- | --- | --- | --- |
|  | **(N=16)** | | **(N=10)** |  |
| LVEDV, mL | 261.6 (194.5–310.7) | | 208.7 (158.7–240.6) | 0.135 |
| LVESV, mL | 206.1 (166.2–242.6) | | 159.9 (115.7–169.8) | 0.060 |
| LVSV, mL | 57.4 ± 19.8 | | 49.8 ± 22.3 | 0.374 |
| LVEF, % | 22.9 ± 8.2 | | 23.6 ± 8.3 | 0.828 |
| RVEDV, mL | 178.7 (151.6–219.0) | | 119.0 (94.2–158.0) | 0.060 |
| RVESV, mL | 118.6 (93.0–165.8) | | 75.2 (61.6–107.9) | 0.041 |
| RVSV, mL | 54.0 ± 19.2 | | 44.4 ± 21.1 | 0.245 |
| RVEF, % | 29.9 ± 10.2 | | 33.7 ± 13.0 | 0.409 |
| LGE burden, % | 34.3 (23.0–52.1) | | 9.7 (7.3–15.8) | <0.001 |
| Native T1, ms | 1384.6 (1363.5–1430.2) | | 1340.1 (1310.1–1406.7) | 0.053 |
| ECV, % | 36.8 (35.3–40.1) | | 33.5 (28.5–35.6) | 0.109 |
| T2, ms | 47.2 (44.9–51.9) | | 43.9 (42.5–45.0) | 0.009 |

Data are expressed as the mean ± standard deviation and median (interquartile range).

CMR, cardiovascular magnetic resonance imaging; CRT, cardiac resynchronisation therapy; ECV, extracellular volume; LBBB, left bundle branch block; LGE, late gadolinium enhancement; LVEDV, end-diastolic volume of left ventricle; LVESV, end-systolic volume of left ventricle; LVSV, left ventricular systolic volume; LVEF, left ventricular ejection fraction; RVEDV, end-diastolic volume of right ventricle; RVESV, end-systolic volume of right ventricle; RVSV, right ventricular systolic volume; RVEF, left ventricular ejection fraction.

**Supplemental Table 8. Predictors of CRT response in patients with LBBB and QRS duration < 150 ms or without LBBB and QRS duration ≥ 150 ms.**

| **Total**  **(N=26)** | **Odds ratio^*^**  **[95% confidence interval]** | ***P*-value** |
| --- | --- | --- |
| **Presence of LGE** | 6.00[1.08-41.51] | 0.049 |
| **LGE burden ≤ 16%**† | 60.0 [6.64–1517.93] | 0.002 |
| **Native T1 ≤ 1328ms**† | N/A | N/A |
| **ECV ≤ 36%**† | 8.80 [1.55–74.71] | 0.023 |
| **T2 ≤ 47ms**† | N/A | N/A |

^*^univariate analysis

†The cut-off values of LGE burden, native T1, and ECV for predicting CRT response identified by an receiver operating characteristic curve analysis in patients with LBBB and QRS duration < 150 ms or without LBBB and QRS duration ≥ 150 ms.

CRT, cardiac resynchronisation therapy; ECV, extracellular volume; LBBB, left bundle branch block; LGE, late gadolinium enhancement; ROC, receiver operating characteristic

**Supplemental Table 9**. **Baseline characteristics according to HF admission, or cardiovascular death**

|  | **Without event** | **With event** | ***P*-value** |
| --- | --- | --- | --- |
|  | **(N=82)** | **(N=19)** |  |
| Age (years) | 65.4 ± 11.4 | 67.4 ± 11.0 | 0.483 |
| Male | 41 (50.0%) | 12 (63.2%) | 0.435 |
| Hypertension | 25 (30.5%) | 6 (31.6%) | 1.000 |
| Diabetes mellitus | 27 (32.9%) | 7 (36.8%) | 0.955 |
| Chronic kidney disease | 10 (12.2%) | 3 (15.8%) | 0.967 |
| AF/AFL | 13 (15.9%) | 8 (42.1%) | 0.026 |
| LBBB | 78 (95.1%) | 10 (52.6%) | <0.001 |
| QRS duration, ms | 165 ± 17 | 161 ± 20 | 0.387 |
| Pre-NYHA classification |  |  | 0.862 |
| 2 | 39 (47.6%) | 8 (42.1%) |  |
| 3 | 43 (52.4%) | 11 (57.9%) |  |
| **Medications** | | | |
| ACEI/ARBs | 23 (28.0%) | 5 (26.3%) | 1.000 |
| Sacubitril/valsartan | 59 (72.0%) | 13 (68.4%) | 0.980 |
| Beta-blockers | 76 (92.7%) | 18 (94.7%) | 1.000 |
| MRAs | 71 (86.6%) | 15 (78.9%) | 0.627 |
| Loop diuretics | 71 (86.6%) | 18 (94.7%) | 0.551 |
| Ivabradine | 26 (31.7%) | 8 (42.1%) | 0.552 |
| SGLT2 inhibitor | 8 (9.8%) | 1 (5.3%) | 0.863 |
| **Echocardiographic parameters** | | | |
| LVESV, mL | 139.3 (112.5–203.5) | 138.7 (110.6–199.0) | 0.893 |
| LVEF, % | 24.5 (20.0–29.0) | 23.0 (20.5–30.5) | 0.876 |
| LAVI, mL/m^2^ | 40.3 (31.7–53.0) | 60.1 (50.4–85.0) | <0.001 |
| RVSP, mmHg | 25.5 (22.0–36.0) | 50.0 (38.3–58.3) | <0.001 |
| E/e’ | 16.0 (11.7–21.4) | 26.3 (19.1–31.2) | 0.008 |
| **CMR parameters** |  |  |  |
| LVEDV, mL | 262.9 (222.1–310.4) | 250.6 (197.0–310.4) | 0.587 |
| LVESV, mL | 201.4 (164.1–250.9) | 183.9 (148.1–243.0) | 0.552 |
| LVSV, mL | 59.4 ± 18.2 | 59.2 ± 21.9 | 0.970 |
| LVEF, % | 23.2 ± 7.9 | 23.7 ± 8.2 | 0.797 |
| RVEDV, mL | 139.6 (112.8–178.5) | 169.3 (131.8–221.8) | 0.073 |
| RVESV, mL | 79.4 (58.1–115.5) | 103.0 (69.1–178.8) | 0.103 |
| RVSV, mL | 55.5 ± 16.7 | 56.5 ± 20.9 | 0.823 |
| RVEF, % | 39.6 ± 13.0 | 35.0 ± 16.8 | 0.192 |
| LGE burden, % | 13.6 (8.9–20.5) | 39.4 (21.7–49.9) | <0.001 |
| Native T1, ms | 1340.2 (1309.3–1390.0) | 1369.6 (1339.1–427.7) | 0.041 |
| ECV, % | 31.4 (28.7–35.3) | 37.0 (35.2–40.3) | <0.001 |
| T2, ms | 42.9 (40.9–44.60) | 45.9 (41.8–48.8) | 0.017 |

Data are expressed as the mean ± standard deviation, median (interquartile range), and absolute numbers (%)

ACEI, angiotensin converting enzyme inhibitor; AF, atrial fibrillation; AFL, atrial flutter; ARB, angiotensin receptor blocker; CMR, cardiac magnetic resonance image; CRT, cardiac resynchronisation therapy; ECV, extracellular volume; eGFR, estimated glomerular filtration rate; HF, heart failure; HT, heart transplantation; LAVI, left atrial volume index; LBBB, left bundle branch block; LGE, late gadolinium enhancement; LVAD, left ventricular assist device; LVEDV, end-diastolic volume of left ventricle; LVEF, left ventricular ejection fraction; LVESV, end-systolic volume of left ventricle; LVSV, left ventricular systolic volume; LVEF, left ventricular ejection fraction; MRAs, mineralocorticoid receptor antagonists; NYHA, New York Heart Association; RVEDV, end-diastolic volume of right ventricle; RVESV, end-systolic volume of right ventricle; RVSP, right ventricular systolic pressure; RVEF, left ventricular ejection fraction; SGLT2, sodium-glucose cotransporter-2

**Supplemental Table 10. Multivariate Cox regression analysis of the HF admission, or cardiovascular death**

| **Total**  **(N=101)** | **Hazard ratio^*^**  **[95% confidence interval]** | ***P*-value** |
| --- | --- | --- |
| **LGE burden > 20%** | 6.73 [1.91–23.79] | 0.003 |
| **Native T1 > 1,333 ms** | 1.15 [0.10–3.31] | 0.795 |
| **ECV > 34 %** | 6.72 [1.40–32.13] | 0.017 |
| **T2 > 45 ms** | 2.08 [0.72–5.96] | 0.175 |

^*^Adjusted by history of atrial fibrillation or atrial flutter, left bundle branch block, left atrium volume index, and right ventricular systolic pressure. Left bundle branch block and right ventricular systolic pressure were found to be significant factors at *P* <0.05 in all models.

ECV, extracellular volume; HF, heart failure; HT, heart transplantation; LGE, late gadolinium enhancement; LVAD, left ventricular assist device

**Supplemental Table 11. Incidence of HF admission, or cardiovascular death in four groups based on the cut-off values of LGE burden and ECV**

|  | **LGE burden ≤ 20%**  **and ECV ≤ 34%**  **(N=46)** | **LGE burden ≤ 20%**  **and ECV > 34%**  **(N=17)** | **LGE burden > 20%**  **and ECV ≤ 34%**  **(N=11)** | **LGE burden > 20%**  **and ECV > 34%**  **(N=25)** | ***P* for trend** |
| --- | --- | --- | --- | --- | --- |
| **Event, n (%)** | 0 (0%) | 4 (23.5%) | 3 (27.3%) | 12 (48.0%) | <0.001 |
| **CRT non-responder, n (%)** | 0 (0%) | 5 (29.4%) | 4 (36.4%) | 15 (60.0%) | <0.001 |

CRT, cardiac resynchronisation therapy; HF, heart failure; HT, heart transplantation; LGE, late gadolinium enhancement; LVAD, left ventricular assist device

**Supplemental Figure 1. Flowchart of the participant selection process**


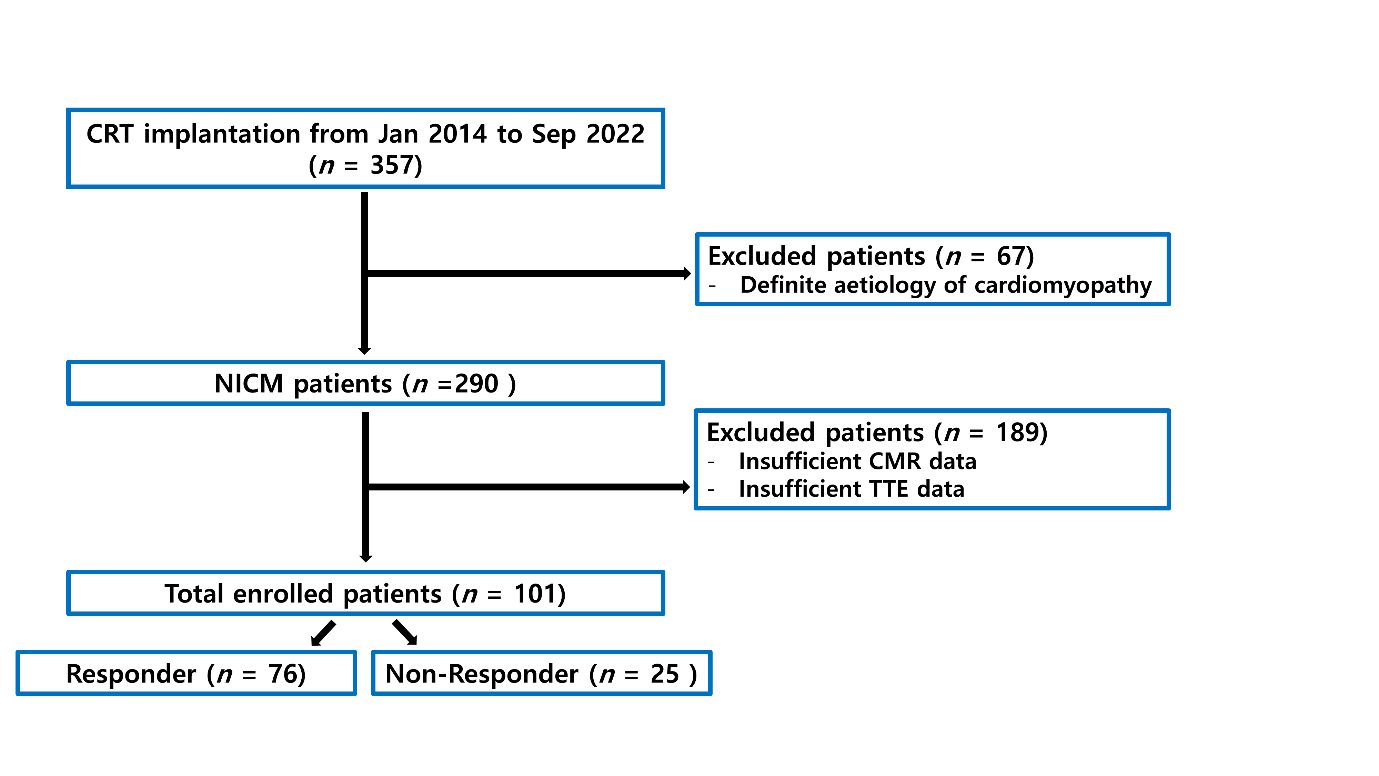


CMR, cardiac magnetic resonance; CRT, cardiac resynchronisation therapy; NICM, non-ischaemic cardiomyopathy

**Supplemental Figure 2. ROC curves displaying the LGE burden and ECV as predictors of CRT response in patients with LBBB and a QRS duration ≥ 150 ms**


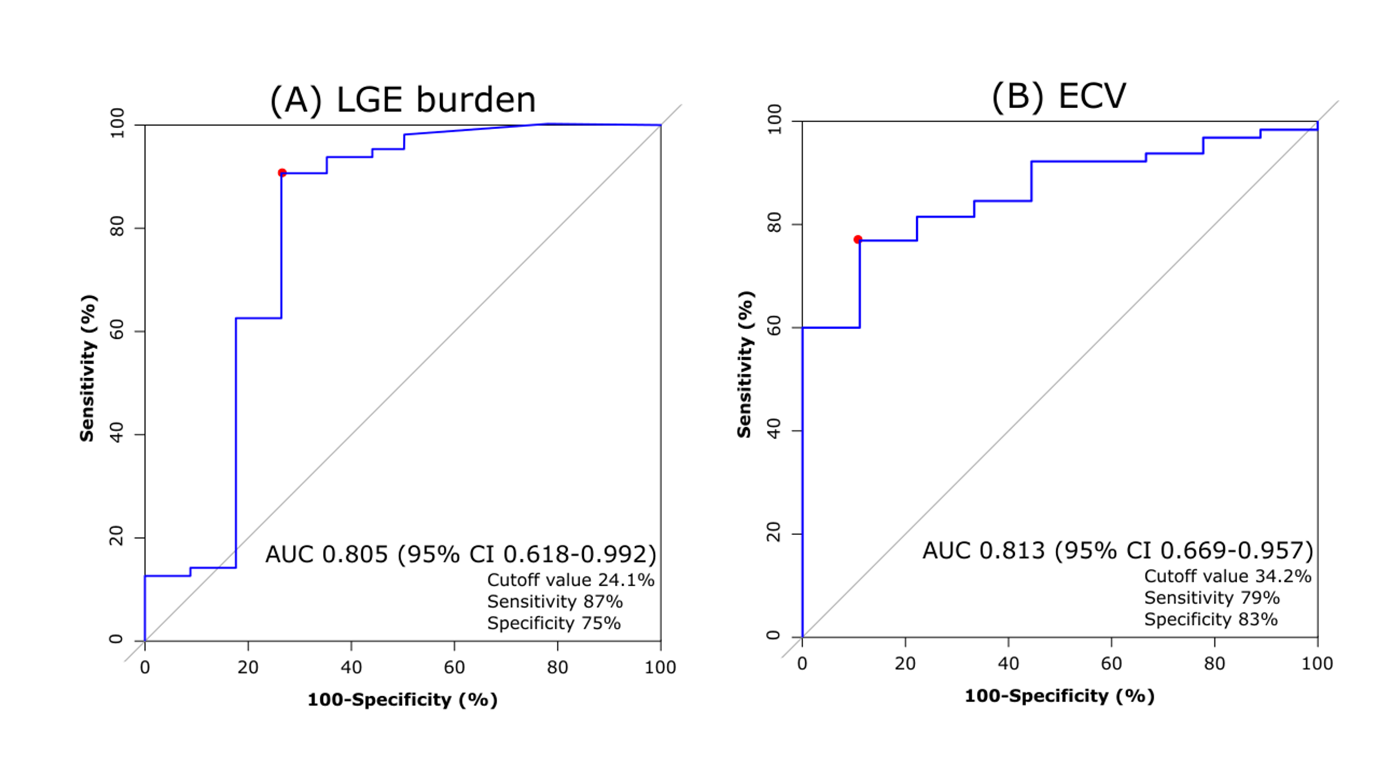


AUC, area under the ROC curve; CI: confidence interval; CRT, cardiac resynchronisation therapy; ECV, extracellular volume; LGE, late gadolinium enhancement; ROC, receiver operating characteristics

**Supplemental Figure 3. Kaplan–Meier curves for HF admission, or cardiovascular death rates according to CRT response**


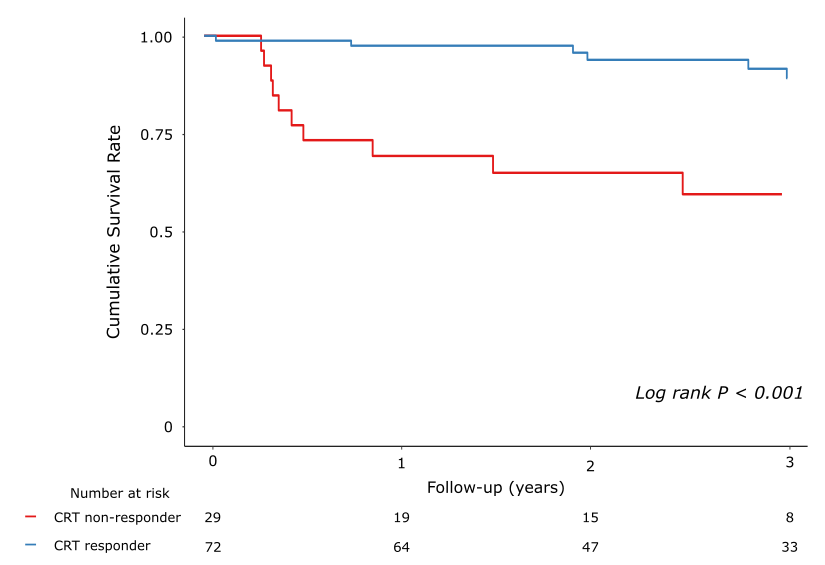


CRT, cardiac resynchronisation therapy, HF, heart failure; HT, heart transplantation; LVAD, left ventricular assist device

**Supplemental Figure 4. Kaplan–Meier curves of HF admission, or cardiovascular death rates in the four groups classified by the cut-off values of LGE burden and ECV**

**
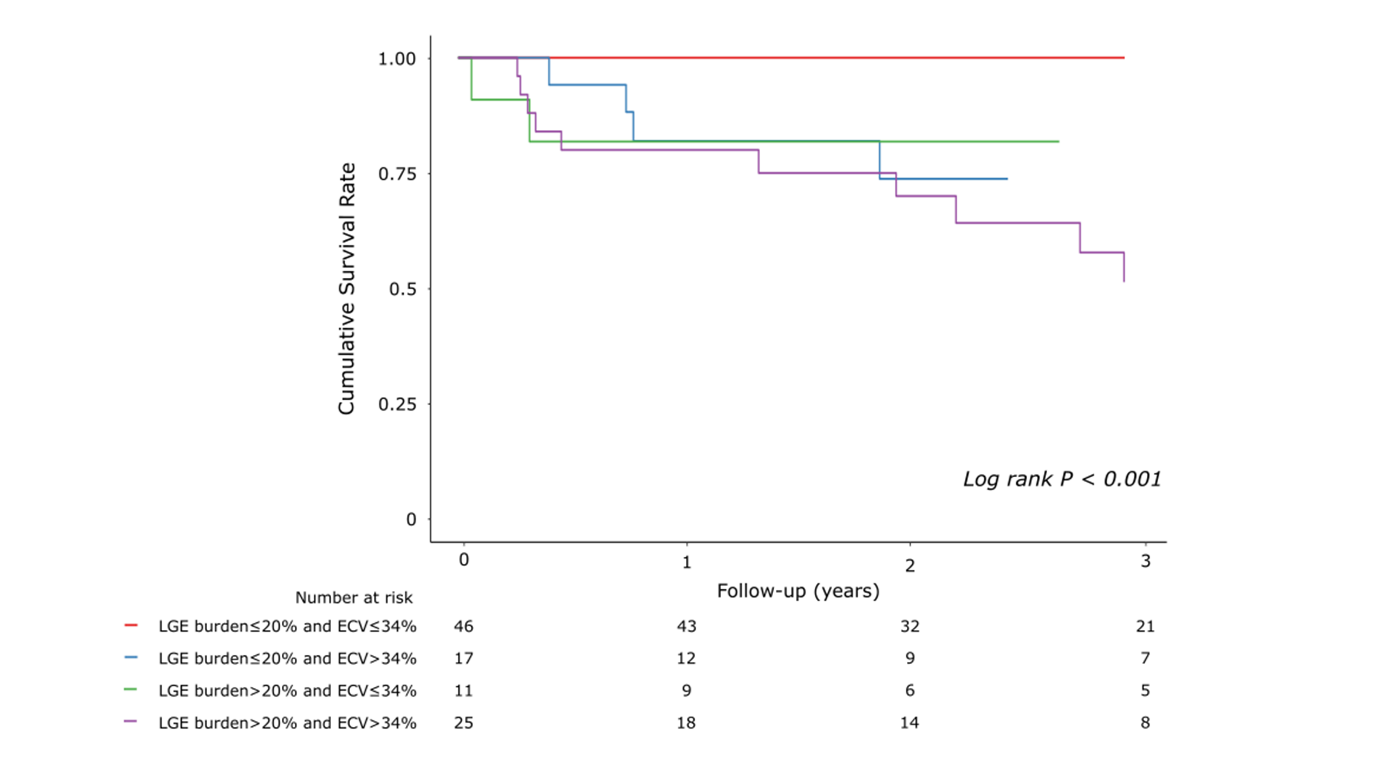
**

ECV, extracellular volume; LGE, late gadolinium enhancement; HF, heart failure; HT, heart transplantation; LVAD, left ventricular assist device
